# *In vivo* Diffusion Tensor Imaging, Diffusion Kurtosis Imaging, and Tractography of a Sciatic Nerve Injury Model in Rat at 9.4T

**DOI:** 10.1038/s41598-018-30961-1

**Published:** 2018-08-27

**Authors:** Gustav Andersson, Greger Orädd, Fahad Sultan, Lev N. Novikov

**Affiliations:** 1 Department of Integrative Medical Biology, Umeå University, Umeå, Sweden; 2 Department of Surgical and Perioperative Science, Section of Hand and Plastic Surgery, Umeå University, Umeå, Sweden; 3 Umeå Center for Comparative Biology (UCCB), Umeå University, Umeå, Sweden

## Abstract

Peripheral nerve injuries result in severe loss of sensory and motor functions in the afflicted limb. There is a lack of standardised models to non-invasively study degeneration, regeneration, and normalisation of neuronal microstructure in peripheral nerves. This study aimed to develop a non-invasive evaluation of peripheral nerve injuries, using diffusion tensor imaging (DTI), diffusion kurtosis imaging (DKI), and tractography on a rat model of sciatic nerve injury. 10 female Sprague Dawley rats were exposed to sciatic nerve neurotmesis and studied using a 9.4 T magnet, by performing DTI and DKI of the sciatic nerve before and 4 weeks after injury. The distal nerve stump showed a decrease in fractional anisotropy (FA), mean kurtosis (MK), axonal water fraction (AWF), and radial and axonal kurtosis (RK, AK) after injury. The proximal stump showed a significant decrease in axial diffusivity (AD) and increase of MK and AK as compared with the uninjured nerve. Both mean diffusivity (MD) and radial diffusivity (RD) increased in the distal stump after injury. Tractography visualised the sciatic nerve and the site of injury, as well as local variations of the diffusion parameters following injury. In summary, the described method detects changes both proximal and distal to the nerve injury.

## Introduction

Peripheral nerve injuries are frequently caused by minor accidents, traumatic injuries, surgery or disease. They often result in severe loss of both sensory and motor functions in the afflicted limb. Although surgical techniques have improved over the last decades, there is still a lack of good clinical outcomes, and some permanent loss in function is often expected following traumatic nerve injury. There is a lack of reliable imaging techniques to specify the type of nerve injury, and to follow regenerating nerves in the clinical setting, but also in preclinical animal models to non-invasively study the regeneration and normalisation of neuronal microstructure in peripheral nerves. These would provide the research community with reproducible models for assessment of novel nerve treatment regimes.

Magnetic resonance imaging (MRI) has become the gold standard for visualisation of the central nervous system, but ultrasound and computer tomography are also important diagnostic tools when it comes to the nervous system as a whole^1^. Following experimental injury, peripheral nerve damage can be visualised on MRI already after 24 hours with a hyperintense T2 signal due to intra- and perineural edema^2^, but diagnostics are primarily based on clinical and electromyographic findings^1^. Development of diffusion-based imaging techniques have been introduced in order to produce *in vivo* reconstruction of peripheral nerves and possibly improve the diagnostic capabilities of MRI^3–5^.

Diffusion tensor imaging (DTI) can be employed to elucidate tissue architecture through the use of directionally varying gradient fields and by processing the recorded signal into a diffusion tensor. The DTI technique is based on signal attenuation derived from the degree of restriction that water molecules are subjected to when diffusing along the axis of the applied gradient. By registering the signal decay from multiple directions, the diffusion in a given tissue, its orientation, and quantitative anisotropy can be modelled^6,7^. Parameters derived from the diffusion tensor include the Fractional Anisotropy (FA); molecular diffusion rate, i.e. the Mean Diffusivity (MD) or Apparent Diffusion Coefficient (ADC); as well as the axial diffusion rate (AD, or λ_//_) and radial diffusion rate (RD, or λ_⊥_). Due to its primarily axonal architecture, water diffusion is anisotropic in healthy nerve fibres^7^, and a multitude of studies have used this fact to attempt to describe pathological changes in the central nervous system, CNS^8–10^ but legitimate concerns have been raised that DTI is incapable of fully distinguishing one type of pathology from another^11,12^ and overinterpretation of what the DTI data represents from a structural or pathological point of view should be avoided^13^. It is thus important to be careful in interpreting the cause of changes to the DTI parameters. A technique that has the potential to further delineate microstructural aspects of CNS white matter is diffusion kurtosis imaging (DKI). DTI is based on the assumption that water diffusion is Gaussian and is thus unable to completely characterise tissue microstructure^12,14^. DKI, on the other hand, calculates parameters while taking into account non-Gaussian diffusion, and may provide more information about heterogeneity of nerve structures^15–19^. DKI has been shown to detect pathological processes where DTI does not, such as increase in mean kurtosis (MK) long after MD changes have normalised^14^. The most common DKI parameters used aside from MK, which corresponds to the average of the diffusion kurtosis along all diffusion directions, is axial kurtosis (AK) and radial kurtosis (RK) which describe the kurtosis along the axial and radial direction of the diffusion ellipsoid respectively^12^. Further DKI data set derived parameters include axonal water fraction (AWF), intra- and extra-axonal diffusivities, and the tortuosity, which can provide detailed information about microstructure of highly aligned fibre bundles^20^.

A further application of DTI and DKI is performing tractography. With this technique it is possible to generate 3D reconstructions of neuronal fibres by use of tracking algorithms based on the calculated diffusion tensors or the kurtosis diffusion orientation distribution function (dODF)^21–24^. DTI tractography has primarily been employed in the CNS in order to describe the neuronal pathways, but it has been shown possible also for peripheral nerves^25^ where it has been used to study different pathologies such as nerve entrapment^3^, traumatic injury^26^ and tumour growth^5^. *Ex vivo* studies have shown that tractography is possible on chemically fixed peripheral nerves^27,28^, and the *in vivo* DTI findings of crush injury has been compared with histology^26,29^ but without tractography. A nerve injury model typically involves the sacrifice of the research animal at each time point of interest in order to study the neuronal regeneration microscopically or by biochemical examinations, but in order to adhere to the principles of ethical animal research, also known as the 3 R’s (Reduce, Replace, Refine), one should constantly strive towards reducing the number of animals and minimise the suffering they endure in all fields of research even further^30^. A non-invasive imaging technique could decrease the number of animals needed to follow degenerative and regenerative changes in peripheral nerves as the same animal can be studied at multiple timepoints, instead of sacrificing animals for histological examination at each timepoint. Furthermore, an imaging protocol minimising scan times would also lower the risk of adverse effects on said research animals as a result of general anaesthesia.

The purpose of this study was to establish an animal model to non-invasively assess degeneration of the sciatic nerves in rat by use of DTI and DKI following neurotmesis. We also wanted to examine the possibility to use DTI-based tractography to visualise the sciatic nerve and the nerve defect following axotomy.

## Results

At 4 weeks post-operatively, all experimental animals displayed visible atrophy of the calf muscles in the operated leg. The DTI scans displayed a clearly discernible sciatic nerve that could be visualised in all three planes (sagittal, coronal and transverse) both preoperatively and at the 4-week scan. No animals were lost during the experiment, nor did they display any adverse effects such as autophagy of the hind limb or infection. The mean SNR was 67.4 ± 11 when positioning the ROI in the uninjured nerve. Positioning the ROI in the distal stump following injury, the SNR was 107.6 ± 11.

**Figure 1:**
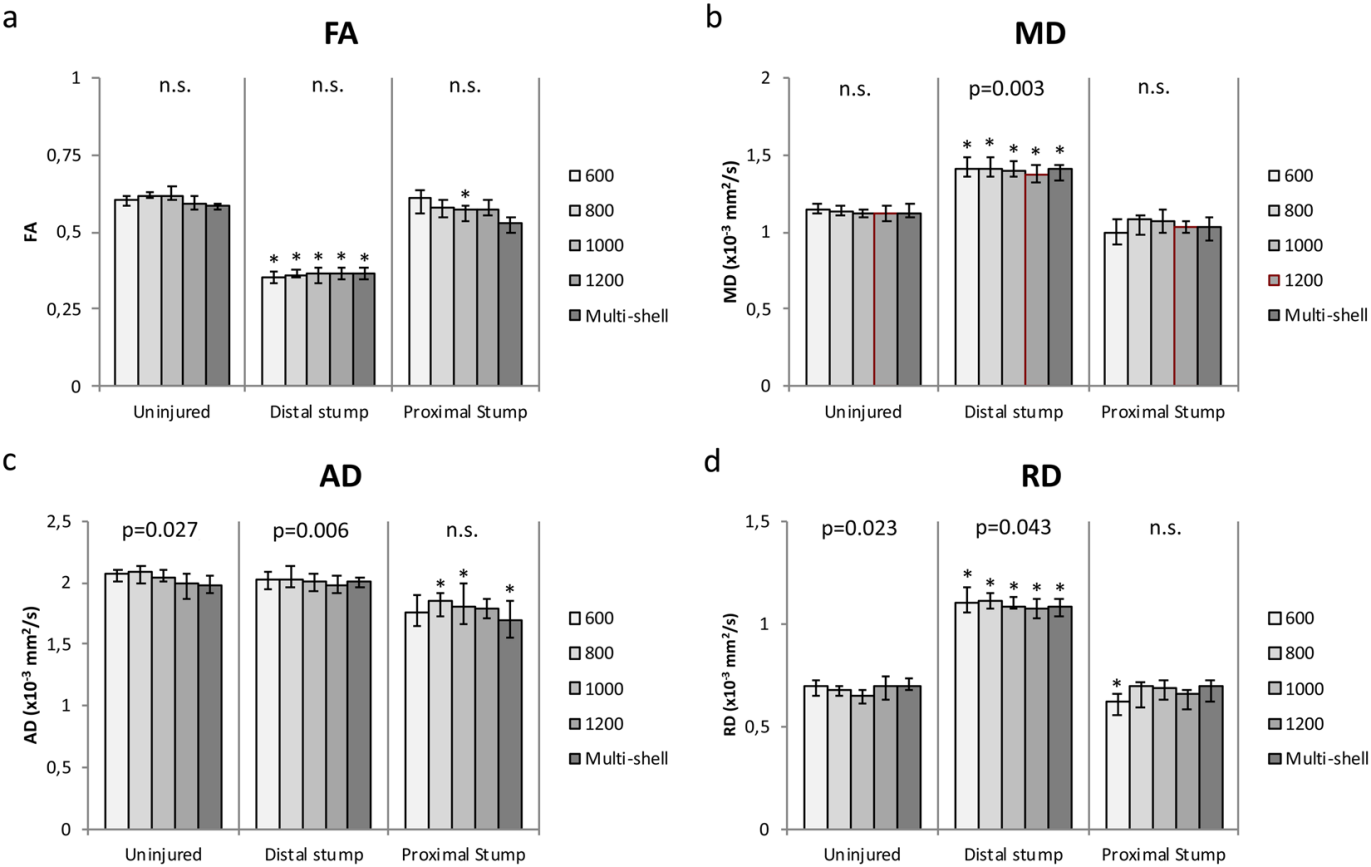
Results of ROI based analysis. Median FA (**a**), MD (**b**), AD (**c**), and RD (**d**) measured in ROI in the uninjured nerve, the distal stump and the proximal stump, at different b-values and of the multi-shell approach. Friedmans Test confirmed that MD, AD, and RD decreases with increasing b-value in the distal stump. The same was seen for AD and RD concerning the uninjured nerve. Error bars indicate the interquartile range of ROI measurements. P-values presented at the top represent Friedmans test of variations within the group. Asterisks indicate a statistically significant difference compared to the uninjured nerve.

### *B-value* impact on diffusion tensor parameters at ROI

Friedmans test showed that there was no significant difference of the estimated FA between the different b-value scans and multi-shell data in the pre-operative scan (p = 0.395), the proximal stump (p = 0.166), or the distal stump (p = 0.620) of the injured sciatic nerve (Fig. 1a). For MD, AD, and RD, there was a trend of decreased parameters at higher b-values in the distal stump which was statistically significant (MD, p = 0.003; AD, p = 0.006; RD, p = 0.043). AD and RD also showed a decrease in the uninjured nerve/pre-operative scan (AD, p = 0.027; RD, p = 0.023) (Fig. 1b).

**Figure 2:**
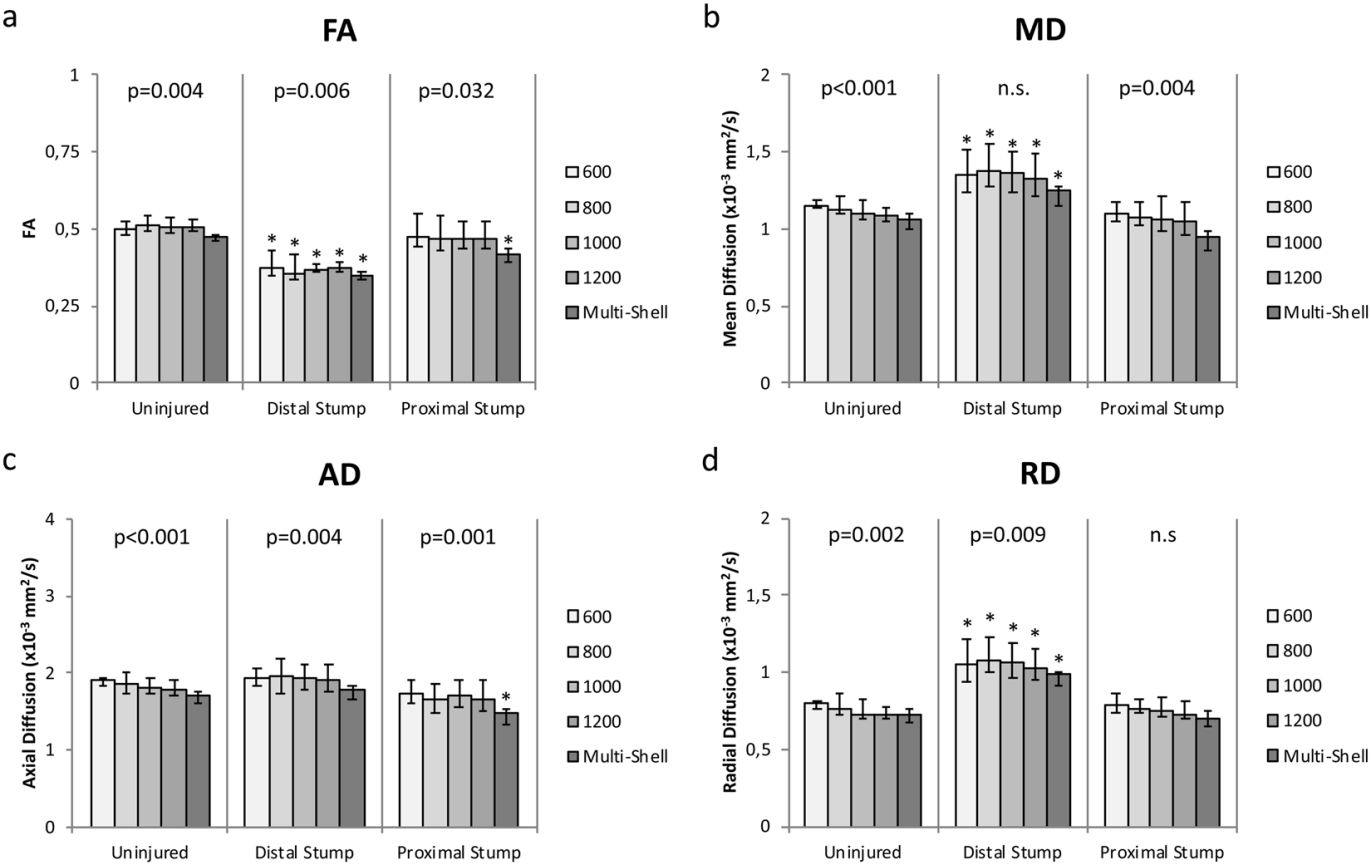
Tractography results. FA (**a**), MD (**b**), AD (**c**), and RD (**d**) of the whole DTI tractography of the uninjured nerve, the distal stump and the proximal stump, at different b-values and of the multi-shell approach. Friedmans Test confirmed that MD, AD, and RD decreases with increasing b-value in all tracts except for the AD in the distal stump. Error bars indicate the interquartile range of ROI measurements. P-values presented at the top represent Friedmans test of variations within the group. Asterisks indicate a statistically significant difference compared to the uninjured nerve.

### Effect of nerve transection on diffusion tensor parameters

Following transection of the sciatic nerve, a significant decrease was seen in the FA of the distal stump as compared to the uninjured nerve (p = 0.015) and the proximal stump (p = 0.015). The median FA was 0.58 (IQR 0.57–0.59) in the uninjured nerves, 0.36 (IQR 0.35–0.38) in the distal stump, and 0.53 (IQR 0.49–0.55) in the proximal stump after nerve injury (Fig. 1a). There was a trend of the FA of the proximal stump being increased compared to the uninjured nerve, but following Bonferroni correction, it was not considered statistically significant (p = 0.063).

The median MD was significantly increased in the distal stump (1.41; IQR 1.33–1.44) following injury as compared to the uninjured nerve (1.12; IQR 1.09–1.18) and the proximal stump (1.03; IQR 0.95–1.10) (Fig. 1b). A trend of decreased MD in the proximal stump was seen, but it was not statistically significant (p = 0.063).

The median AD was significantly decreased in the proximal stump (1.70; IQR 1.55–1.85) after injury when compared with the uninjured nerve (1.98; IQR 1.91–2.06) and the distal stump (2.00; IQR 1.96–2.04), respectively. However, no significant differences were noted in the distal stump as compared to the uninjured nerve (Fig. 1c).

The median RD was significantly increased in the distal stump (1.09; IQR 1.04–1.12) after injury when compared with the uninjured nerve (0.69; IQR 0.68–0.73) and the proximal stump (0.70; IQR 0.62–0.73), respectively. However, no significant differences were noted between the proximal stump and the uninjured nerve (Fig. 1d).

All these changes were also observed when the individual b-values were used instead of the multi-shell approach, but with some notable differences. The slight FA-value decrease in the proximal stump (0.57; IQR 0.54–0.58) after injury was statistically significant compared to the uninjured nerve (0.61; IQR 0.60–0.65) when using a b-value of 1000 s/mm2 (p = 0.027). Further, the difference in AD-value between the distal (1.41; IQR 1.36–1.48) and proximal stump (1.08; IQR 0.99–1.10) following injury was only statistically significant at b-values of 800 s/mm2 (p = 0.027) and 1000 s/mm2 (p = 0.050). Using a b-value of 600 s/mm2 allowed the detection of a significant difference (p = 0.039) in RD between the proximal stump (0.62; IQR 0.55–0.66) and the uninjured nerve (0.70; IQR 0.65–0.73), which was not seen in other b-values or when the multi-shell approach was used.

### Tractography

Tractography based on the DTI parameters defined in the Materials and Methods section showed a significant difference of the averaged FA of the whole tract for the different b-values and multi-shell approach in the preoperative scans (p = 0.004), the proximal stump (p = 0.006), and the distal stump following nerve injury (p = 0.032), where the multi-shell displayed the lowest FA value. The MD, AD, and RD showed a trend of decreasing at higher b-values compared to the lower b-values (Fig. 2), which was statistically significant for both preoperative scans, as well as the proximal stump, with the exception of MD for the distal stump and RD for the proximal stump.

When comparing the tracts of the uninjured nerve, the proximal stump, and the distal stump, the same relative changes of the diffusion parameters as seen in the voxel-based analysis were detected (Fig. 2), with the exception of the multi-shell approach which showed a statistically significant decrease in the proximal stump as compared to the uninjured nerve, while none of the individual b-values did.

**Figure 3:**
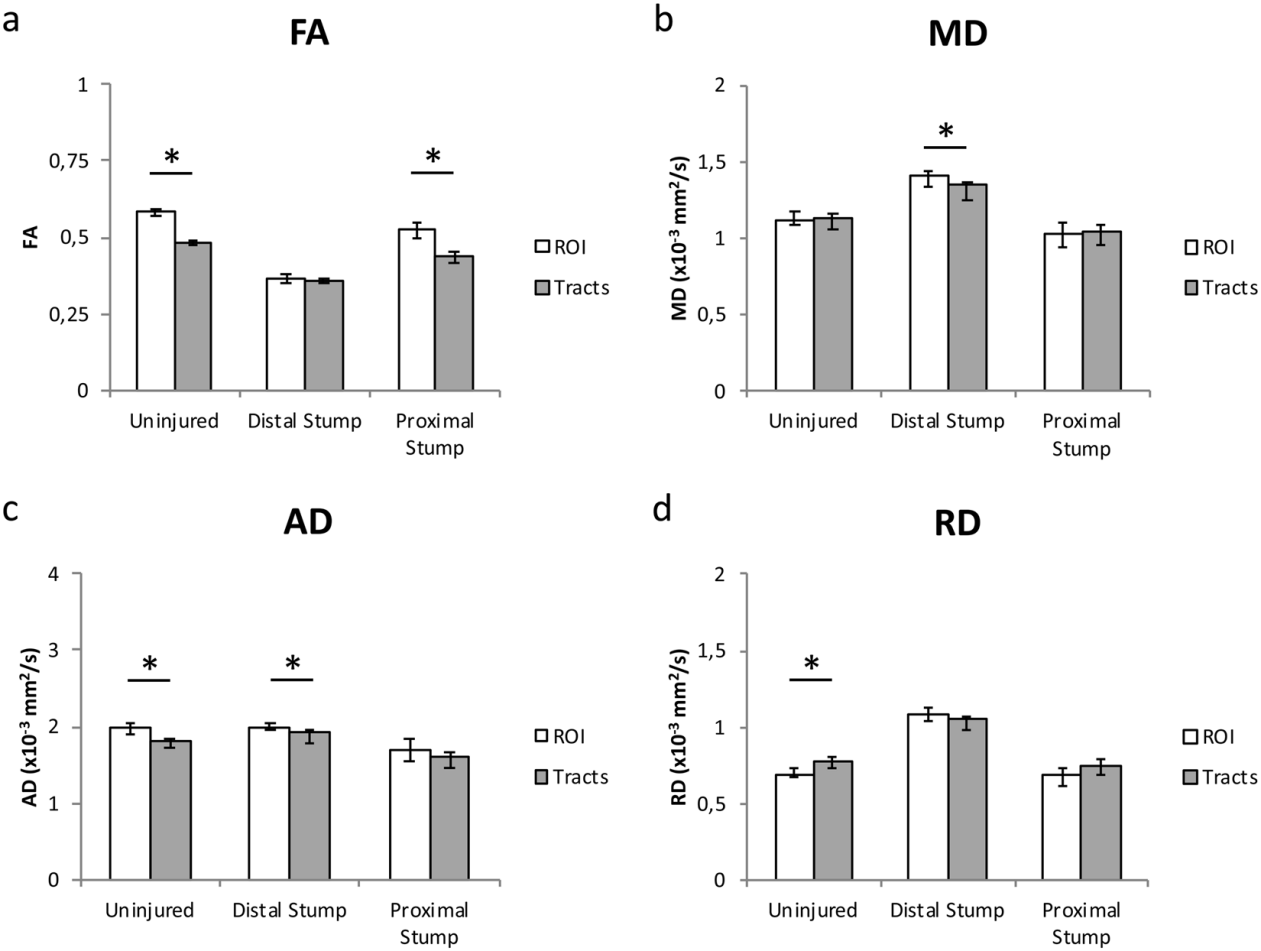
Comparison of ROI analysis and tractography. Comparison of multi-shell FA (**a**), MD (**b**), AD (**c**) and RD (**d**) between ROI measurement and tractography. A statistically significant decrease was seen in the tractography compared to the ROI measurement in the uninjured nerve for FA, and AD whereas RD was higher in the tractography. The MD and AD was lower in the distal stump in the tractography, and the same was seen for FA in the proximal stump. Error bar indicate the interquartile range of ROI measurements.

The FA’s of the tracts were lower than that of the ROI-based analysis, except for in the distal stump where no significant difference was seen (Fig. 3a). The MD values of the tracts were lower than in the ROI for the distal stump (Fig. 3b), and AD was significantly higher in the voxel-based analysis, except for in the proximal stump where the difference was not statistically significant (p = 0.051) (Fig. 3c). RD was significantly higher in the tracts in the uninjured nerve, but not in the proximal or distal stump (Fig. 3d).

**Figure 4:**
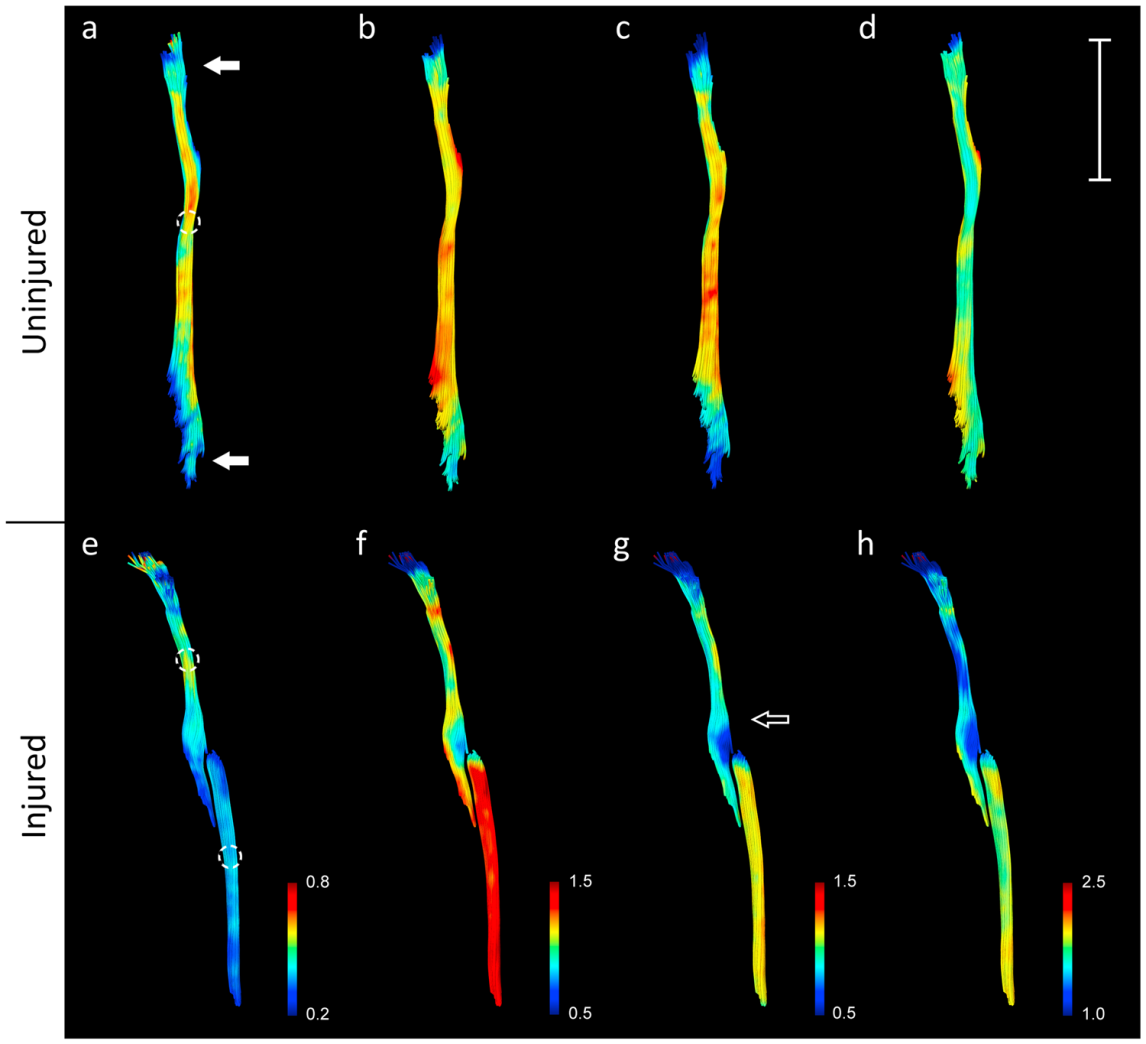
Tractography rendered based on the DTI indices. Local variations in DTI indices along the nerve following injury (**e**–**h**) in the sciatic nerve of one the experimental animals. The tractography displayed lower FA (**a**,**e**), MD (**b**,**f**), RD (**c**,**g**), and (**d**,**h**) at the edge of the coil (a, solid arrow) in both injured and uninjured nerves. Marked variations in DTI measurements could be detected locally compared to the rest of the nerve (hollow arrow). Circles indicate positioning of ROIs. Bar = 10 mm.

**Figure 5:**
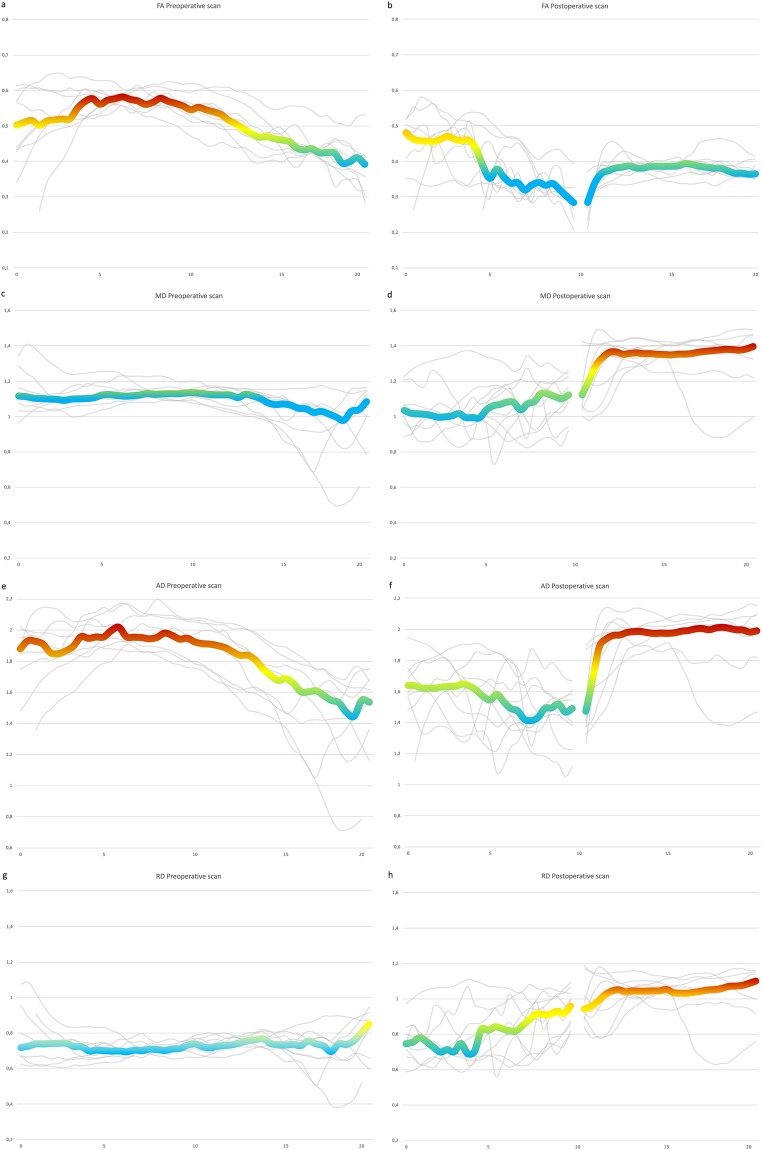
Tract Analysis Report of the preoperative and postoperative tractographies. The colored line represents the median value of all research animals. The grey lines represent the individual research animals tractography analysis report. Close to the edges of the coils receptive field the parameter estimates appear less precise, and the same can be seen when approaching the nerve bifurcation. FA decreases when approaching the nerve injury (**b**). MD increases distally of the nerve injury (**d**). AD is lower in the proximal stump than the uninjured nerve but is higher in the distal stump (**e** & **f**). RD increase when approaching the nerve injury and is further increased in the distal stump (**h**). Y-axis represents the individual DTI indices. X-axis represents length of tracts in mm.

By rendering the tracts with a colour gradient based on the diffusion tensor parameters, the most proximal and distal part of the tracts, which were close to the edge of the receptive field of the surface coil, displayed a drop in the diffusion parameters (Fig. 4a). The tractography also showed a variation in the diffusion indices at different points along the injured nerves, with marked changes at the distal end of the proximal stump (Fig. 4g). In the tract analysis based on the multi-shell tractographies, the same trend could be seen with drop in diffusion parameters closer to the edge of the receptive field (Fig. 5), and when approaching the nerve bifurcation into the tibial and peroneal nerves. Variations in the proximal stump following injury was very evident between individuals (Fig. 5b,d,f,h).

### Effect of nerve transection on kurtosis imaging parameters

**Figure 6:**
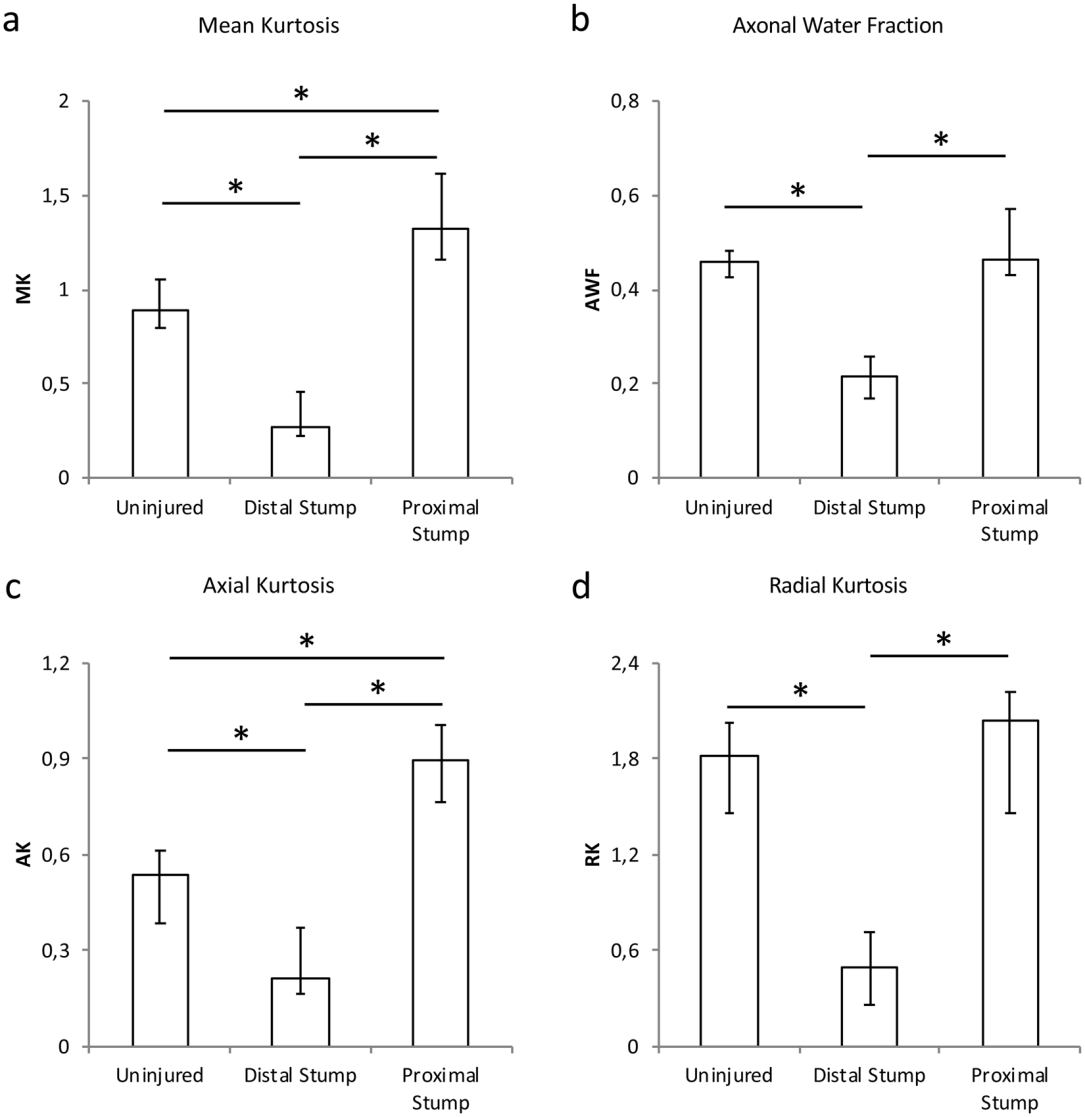
Diffusion Kurtosis Imaging results. MK shows a statistically significant decrease in the distal stump and increase in the proximal stump following injury. AWF is decreased in the distal stump. AK is decreased in distal stump and increased in the proximal stump. RK is decreased in the distal stump following injury.

Following injury, the MK increased in the proximal stump (1.33; IQR 1.16–1.61) and decreased in the distal stump (0.27; IQR 0.23–0.46) as compared to the uninjured nerve (0.89; IQR 0.80-0.11) (Fig. 6a). The same was seen for axonal kurtosis (AK) where the proximal stump (0.89; IQR 0.77–1.01) showed a statistically significant increase over the uninjured nerve (0.54; IQR 0.38–0.61), as well as a significant decrease in the distal stump (0.21; IQR 0.17–0.37) (Fig. 6c).

AWF was significantly decreased in the distal stump (0.21; IQR 0.17–0.26) compared to the proximal stump (0.46; IQR 0.43–0.57) and the uninjured nerve (0.46; IQR 0.43–0.48) (Fig. 6b). RK was significantly decreased in the distal stump (0.50; IQR 0.26–0.72) compared to the proximal stump (2.05; IQR 1.46–2.22) and the uninjured nerve (1.82; IQR 1.47–2.02) (Fig. 6d).

## Discussion

In previous studies, peripheral nerves have been visualised using diffusion tensor imaging and tractography^3,5,25,31^. Most DTI studies have used CNS as a model, and a standardised *in vivo* model of peripheral nerve injury is yet to be established although some examples have been described^26,29^. In order to develop the foundation for clinically applicable DTI techniques for peripheral nerve injury, and to follow the principles of the 3 R’s of animal research, we used a protocol designed to minimize scan time but still produce reliable results for detection of structural changes in the injured nerves. Furthermore, using this technique instead of histological studies at multiple time-points will drastically decrease the total number of animals needed in peripiheral nerve research as the same animal can be scanned repeatedly.

Although DTI is considered less sensitive in detecting crossing fibres of neural tissue in the CNS compared to other high-order diffusion methods such as high angular resolution diffusion imaging (HARDI)^17,32–34^, due to the basic architecture of peripheral nerves with mainly longitudinally aligned fibres outside the branching points, one would expect it to be sufficient for performing tractography. A combination of tractography and placement of ROI’s to calculate FA, MD, AD, and RD, could provide information about not only the localisation of injury, but also the directions and the extent of subsequent nerve regeneration. Whether DTI is sensitive enough to detect certain characteristics of regenerating nerve tissue such as myelination and edema has been investigated^35–37^, and it is becoming all the more evident that DTI parameters are not sensitive enough to detect specific biological processes (i.e. inflammation, demyelination etc)^12^. Here, DKI has proved more promising as it is less sensitive to interference by local edema^12^ and has also shown increased sensitivity in detecting certain pathological conditions in the central nervous system^38^ as compared to DTI. Our results suggest that information concerning FA of the structures of interest can be detected at the entire range of b-values (600–1200 s/mm2), but the minute changes in the proximal stump were only statistically significant at 1000 s/mm2, whereas proximal stump changes of MD, AD and RD where most readily detected at 600 s/mm2. These variations may be indicative of what b-value range to study the proximal stump, and in clinical cases where the MRI scan does not visualise the site of peripheral nerve injury, a decrease in AD could be indicative of a more distal injury. AD decrease has previously been seen in cases of axonal injury^34^ which would be expected in this injury model as well. This, however, needs to be validated in future studies, and one major concern may be the result of the tensor model being misinterpreted due to inflammatory reactions in the area of interest. It has been shown that AD will decrease as a result of increased cellularity, and RD and AD both will be affected by vasogenic edema^39^. However, our DKI results also indicate changes occurring in the proximal stump following the nerve injury as evident by the rise in MK which would imply increase in tissue heterogeneity^15^. As MK is less prone to be affected by cellular infiltration and edema than traditional DTI^12^ the extra scan time for obtaining the extra b-values needed for diffusion kurtosis estimation can be motivated. The increase in AK of the proximal stump may be related to changes to the intra-cellular structures, whereas changes to RK which was most marked in the distal stump is considered to be more influenced by cellular membranes and myelin^12^, which are expected to be disrupted following Wallerian degeneration^40^ of the distal nerve stump, and thus resulting in a lower RK.

For the purpose of this study, we chose to create a nerve lesion using sharp transection at the midpoint between the sciatic notch and the distal nerve bifurcation into major branches to be able to clearly visualise it using DTI based tractography as this would be on a relative “straight” course of the nerve in the thigh of the rat. This type of injury is known to cause Wallerian degeneration of the distal stump, with demyelination^41^. This study did not aim to study the functional outcome of the denervation, nor the degree of regeneration, but rather the *in vivo* application of DTI, DKI, and tractography on the peripheral nerve following injury.

Pilot studies showed that motion artefacts and ghosting was a complicating factor even though the animal was sufficiently sedated. DTI is known for its susceptibility to motion artefacts and eddy current artefacts^42^. Respiratory and/or cardiac gating may have alleviated some of these issues but would also increase the scan time markedly. We therefore designed the setup in such a way that it helped to decrease any motion artefacts. The sciatic nerve model was selected since it is localised far away from the respiratory tract and the heart, and there are no directly neighbouring major vessels, which minimises the negative impact of these structures on the scans. Parallel imaging (GRAPPA) and ghost correction was used when performing the scans, and post-processing with motion correction and eddy current correction, was also employed to minimize any artefacts commonly seen in DTI scans^42^.

The current study demonstrates that structural changes can be detected with DTI and DKI not only in the distal nerve stump undergoing Wallerian degeneration, including demyelination, but also in the proximal stump where one would expect axonal dieback, cellular infiltration and sprouting of nerve fibres^41^. Whether or not the detected parameter changes relate to these specific processes cannot be answered by DTI alone, but of note was the finding that AD decreased in the proximal stump after 4 weeks when compared with the uninjured nerve, indicating that DTI can detect the reactive processes in this area and not only in the distal nerve stump^41^. Looking at our results it is evident that tractography helps visualise the characteristics at different levels in the nerve. Although there were statistically significant changes at the position of the ROI, the Tract Analysis Report showed that these changes may be more evident closer to the injury site, and it is therefore important to look at the diffusion-based parameters over the whole nerve in order to not misinterpret the results. It was also evident that the diffusion imaging is affected closer to the edges of the coils receptive field, likely due to increased inhomogeneity of the magnetic field and/or lower signal^42^.

Although there were statistically significant differences between certain b-values for MD, AD, and RD, the practical importance of these minute differences can be questioned. These changes were not large enough to lead to misinterpretation of the integrity of the nerve.

However, the variability in results with different b-values in this study may be of importance when looking for small changes in peripheral nerves, as certain b-values detected statistically significant changes in the diffusion parameters that were not seen in others, but the added value of performing DKI is likely to outperform DTI parameter analysis. Previous studies have suggested that an optimal b-value for visualising peripheral nerves using traditional DTI is 800 s/mm2^3,26^. DKI requires multiple b-values, but the minimum requirement suggested is two b-values and 15 directions^43^, which should not result in unreasonably long scan times.

This study is limited in that we chose a narrow range of b-values, mainly because this range is most commonly employed in clinical settings for DTI^42^, but it may be considered a weakness of this study to not include a higher b-value for the DK estimation, as a combination of 1000 and 2000 s/mm2 has been suggested as an ideal setting for DKI in clinical use^43^. Furthermore, the use of the FA-map when positioning the ROI could potentially be problematic in case of nerve deterioration to the degree of showing a similar FA value as that of the surrounding tissues. In this study it did not present any issues, as the nerve, including the distal stump, was clearly visible (see “Supplementary Information Fig. 1”), but using an alternative type of scan may serve as a better map to position the ROIs. Furthermore, the use of single ROIs in the analysis of proximal and distal stump after surgery does not give a complete view of the varying diffusivities along the tract, nor does an average of the whole tract either, as was evident by the tract analysis report.

In conclusion, with this study we set out to establish a diffusion-based model of peripheral nerve injury detection. We used a sciatic nerve injury model in rat and utilized a 9.4 T Bruker BioSpec 94/20 USR system to collect diffusion-weighted images to calculate the diffusion tensors and kurtosis parameters in the region of interest. The positioning of the experimental animal and scan parameters employed is described, and the effects upon diffusion and kurtosis parameters of interest 4 weeks following nerve injury are presented.

Further improvements to the protocol can be made with adapting the number of directions and choice of b-values, however changes in both the proximal and distal stump can be detected using the present setup. Further studies are required to specify the gradual changes of degeneration and possible reinnervation of the distal stump following injury as seen by DTI and DKI, but this setup should provide a good tool for non-invasive evaluation of these aspects.

## Methods

### Experimental animals and surgery

The experiments were performed on adult (10–14 weeks old, n = 10) female Sprague-Dawley rats (Charles River Laboratories). The animal care and experimental procedures were carried out in accordance with Directive 2010/63/EU of the European Parliament and of the Council on the protection of animals used for scientific purposes and were also approved by the Northern Swedish Committee for Ethics in Animal Experiments (No. A50-13). All surgical procedures were performed under general anaesthesia using isoflurane (Attane vet, 1000 mg/g, Oiramal Healthcare, UK). After surgery, the rats were given the analgesic Finadyne (Schering-Plough, Denmark; 2.5 mg/kg, s.c.).

**Figure 7:**
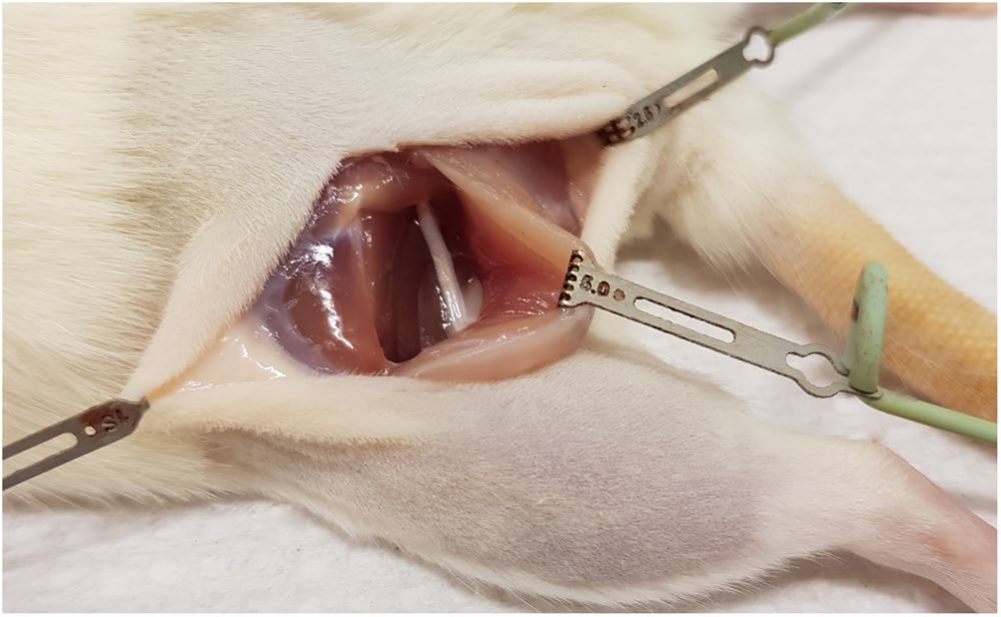
Surgically exposed sciatic nerve in left hind limb of rat.

Under sterile conditions the left sciatic nerve was exposed (Fig. 7) and cut with a pair of straight micro scissors at a point 5 mm distal to the sciatic notch.

The proximal nerve stump was covered with a thin layer of Spongostan® (Johnson & Johnson Medicals, UK) to prevent spontaneous nerve regeneration into the distal nerve stump, and the muscles and skin were closed in layers.

### *In vivo* imaging

MRI scans were performed at two time points; pre-operatively and 4 weeks post-operatively on the same animals. The animals were scanned using a 9.4 T Bruker BioSpec 94/20 USR system connected to a rat brain array coil combined with a 87 mm QUAD resonator coil and running ParaVison® 6.1 software (Bruker Biospin Group, Bruker Corporations, Germany) under isoflurane anaesthesia. Respiration was monitored using a respiration pillow (SA Instruments Inc., Stony Brook, USA).

The animals were positioned in a right lateral decubitus position with the brain array coil positioned over the left hip and thigh. A custom-made foam support was placed between the legs of the animal to minimize unwanted motion during the scans, and to achieve stable contact between the coil and the animal. Markings were made on the foam support in order to allow the animal and coil positioning to be recreated for all scans (see Supplementary Information Fig. 2). In order to minimize motion from abdominal breathing, a 2 cm wide strap was placed directly proximal of the surface coil to stabilise the distal abdomen and pelvis of the animal.

An initial scan was performed to establish the position of the animal and localise the area of interest. The B0 field map was calculated before running the DTI scan in order to perform shimming. The data was collected using a DTI EPI Spin Echo diffusion scheme with a total of 32 diffusion-sampling directions acquired in 100 axial slices. In total, 6 b = 0-images were collected. The in-plane resolution and slice thickness were 0.4 mm in order to achieve isotropic voxels. TE = 22 ms, TR = 8000 ms, no averaging. FOV 38.4 mm, matrix size 96 × 96. A DTI scan with the b-value set to 600, 800, 1000, and 1200 s/mm2 was collected. Segmented k-space acquisition of 3 readout segments was utilized. Navigator, Automatic Ghost Correction and Grappa Multishot Adjustment was activated. The diffusion time was 10.5 ms. The diffusion encoding duration was 4.5 ms. Saturation slabs were positioned medial to the thigh to cover bladder, intestines, tail and any other air pockets, in order to minimise artefacts. Each DTI scan took 15 min, 12 secs for a total of approximately 61 minutes for all four b-value scans.

Post-processing, tensor calculation and analysis of data was performed in DSI-studio (Dec 7 2016 Build). DSI-Studio employs the same tensor calculation as is described by Jiang *et al*.^44^. The diffusion weighted image data was processed with Motion Correction by registering the b0 images to the first b0 image to create a transformation matrix which is then applied to each DWI, and Eddy Current correction which registers DWI to the b0 and applies the registration matrix to the DWI’s. The images were up-sampled with a factor of 2 through linear interpolation in order to facilitate the placement of ROI’s^45^. A multi-shell diffusion scheme was employed were the four b-values were combined to produce a fifth DTI data file for each scan. One scan in the preoperative group was excluded from this as the Motion correction was not able to correct for severe translocation in the collected DWI’s. This was the result of the scan having to be restarted to collect the last two b-values, and the animal repositioned due to scanner malfunction at the time of scanning.

**Figure 8:**
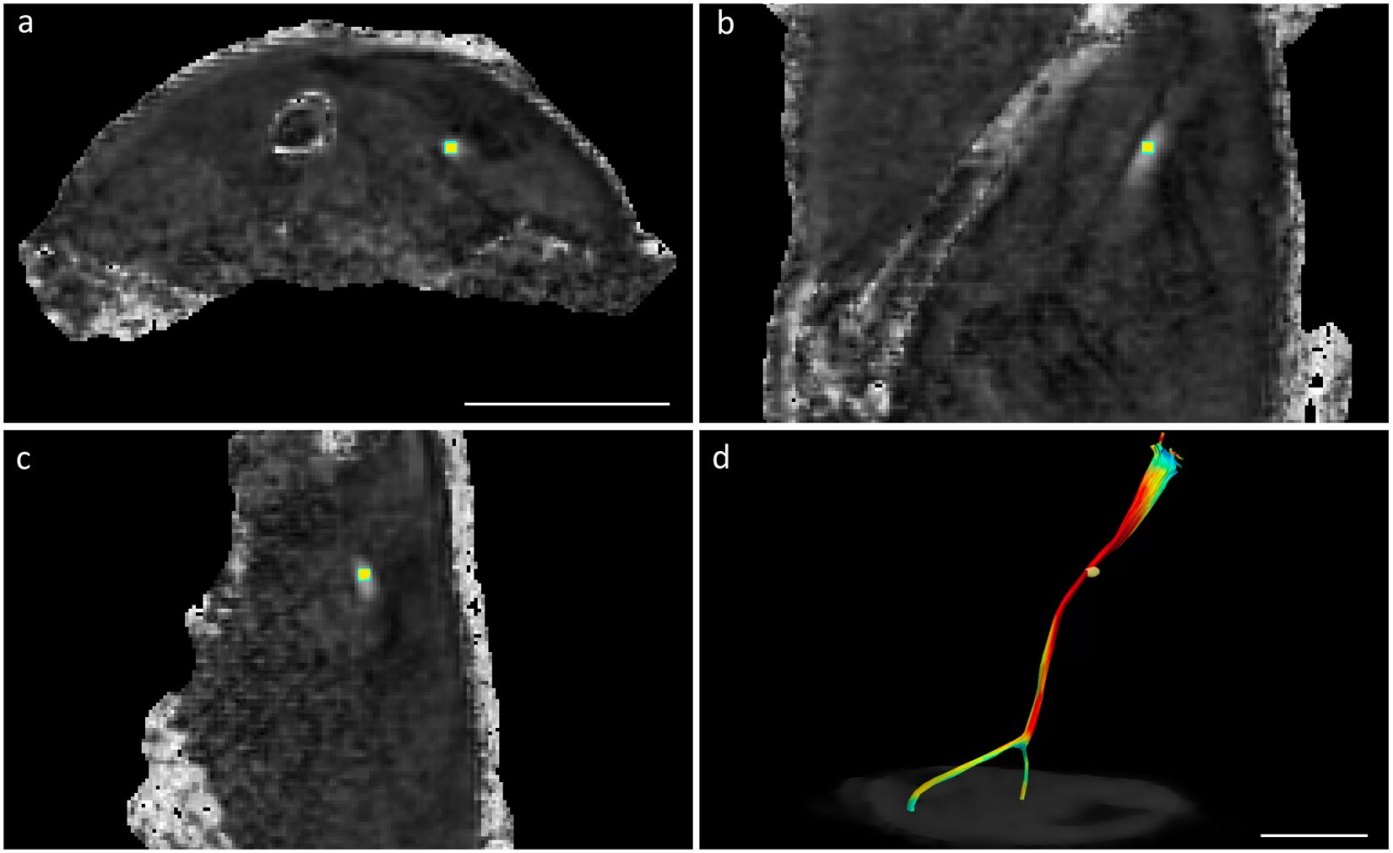
Greyscale FA map with ROI positioned in the sciatic nerve. Axial (**a**), sagittal (**b**) and frontal views (**c**) were used to verify the placement of ROI. *In vivo* tractography of the uninjured sciatic nerve in rat is shown in (**d**). Bar = 10 mm. For FA maps from additional subjects, see “Supplementary Information Fig. 1”.

A spherical ROI consisting of a total of 19 voxels was manually placed at 10 mm proximal to the sciatic nerve bifurcation into tibial and fibular nerves in the pre-operative scans. In the post-operative scans, the ROIs were placed at 5 mm proximal and at 5 mm distal to the transection site. The reconstructed fractional anisotropy map was used to position the ROI. All three viewports (axial, sagittal, coronal) were checked to make sure the ROI was placed in the centre of the nerve, and that it did not extend outside the nerve tissue (Fig. 8). The same ROI-position (XYZ-coordinate) was used for all four different b-value scans and the multi-shell data. The position was manually controlled to verify that the localisation was the same following post-processing, as one scan had been performed with repositioning of the animal which was too large for the motion correction to correct for and manual correction of the ROI placement was required. The ROI was analysed for fractional anisotropy (FA), mean diffusivity (MD), axial diffusivity (AD), and radial diffusivity (RD), and the averaged value (i.e. the mean) of all the voxels in the ROI was used to perform the subsequent statistical analysis.

Tractography was performed for all b-values (600, 800, 1000, and 1200 s/mm2) and the multi-shell data separately using a deterministic fibre tracking algorithm^46^ of all fibres passing through the ROI’s described in earlier text. Angular threshold was set at 30 degrees as our pilot studies showed minimal changes in tracking results in the range 30–60 degrees, but a clear decrease in track length with lower angular thresholds <30, and an increase of anatomically incorrect tracts at higher thresholds. Step size was 0.4 mm and the anisotropy threshold was set at 0.3. Tracts shorter than 2 mm were discarded. The visualised tracts were rendered to display each diffusion parameter separately with a colour gradient. The averaged (mean) diffusion parameters of the entire tracts were used for statistical analysis. A tract analysis report was also produced to plot any local variations in the diffusion tensor parameters by recording the diffusion tensor indices along the rendered tracts of the multi-shell derived FA maps. Kurtosis tensor estimation was performed using the protocol described by Veraart *et al*.^47^. The four b-value scans described in earlier text (600, 800, 1000, 1200 s/mm2, 32 directions) were used. Further white matter metrics were determined using the method described by Fieremans *et al*.^48^. Mean kurtosis and axonal water fraction was subsequently analysed in DSI-studio by the placement of ROIs in the same localisations as the DTI analysis.

In order to determine the signal-to-noise ratio (SNR), a numerical value was computed using two sequential observations of b0 images in both the preoperative and postoperative scans. An averaged image and a difference image of the two observations were computed using ImageJ (version 1.52a), then the SNR was computed as the mean value of a ROI positioned at the area of interest (the sciatic nerve) in the average image divided by the standard deviation over the voxels in the same ROI within the difference image^49^.

### Statistical analysis

Non-parametric statistics was used. A p-value of < 0.05 was considered significant. The diffusion tensor parameters (FA, MD, AD, RD) in the non-operated sciatic nerve, and the proximal and distal stump in the transected nerve, were compared between the different b-values and the multi-shell data using Friedmans Test. Wilcoxon Signed Ranks test was used to detect any differences in diffusion and kurtosis parameters (FA, MD, AD, RD, MK, AWF) between the pre-operative scan, and the post-operative scans of the proximal and distal stump by pairwise comparisons. Bonferroni correction was used to correct for the multiple comparisons, and the presented p-values are given in their corrected form. The presented values of the statistical analysis are medians and interquartile range. IBM SPSS Statistics 24 (9.6.0.0) was used to perform all statistical calculations.

## Electronic supplementary material

Supplementary Information

## Data Availability

The data (DTI-scans) generated and analysed during the current study are available from the corresponding author on reasonable request.

## References

[1] Ohana M (2014). Current and future imaging of the peripheral nervous system. Diagn. Interv. Imaging.

[2] Stanisz GJ, Midha R, Munro CA, Henkelman RM (2001). MR properties of rat sciatic nerve following trauma. Magn. Reson. Med..

[3] Stein D (2009). Diffusion tensor imaging of the median nerve in healthy and carpal tunnel syndrome subjects. J. Magn. Reson. Imaging.

[4] Takahara T (2008). Diffusion-weighted MR neurography of the brachial plexus: feasibility study. Radiology.

[5] Vargas MI, Viallon M, Nguyen D, Delavelle J, Becker M (2010). Diffusion tensor imaging (DTI) and tractography of the brachial plexus: feasibility and initial experience in neoplastic conditions. Neuroradiology.

[6] Chenevert TL, Brunberg JA, Pipe JG (1990). Anisotropic diffusion in human white matter: demonstration with MR techniques *in vivo*. Radiology.

[7] Beaulieu C (2002). The basis of anisotropic water diffusion in the nervous system - a technical review. NMR Biomed..

[8] Song SK (2002). Dysmyelination revealed through MRI as increased radial (but unchanged axial) diffusion of water. Neuroimage.

[9] White T, Nelson M, Lim KO (2008). Diffusion tensor imaging in psychiatric disorders. Top. Magn. Reson. Imaging.

[10] Budde MD, Xie M, Cross AH, Song SK (2009). Axial diffusivity is the primary correlate of axonal injury in the experimental autoimmune encephalomyelitis spinal cord: a quantitative pixelwise analysis. J. Neurosci..

[11] Sun SW (2006). Noninvasive detection of cuprizone induced axonal damage and demyelination in the mouse corpus callosum. Magn. Reson. Med..

[12] Steven AJ, Zhuo J, Melhem ER (2014). Diffusion kurtosis imaging: an emerging technique for evaluating the microstructural environment of the brain. AJR Am. J. Roentgenol..

[13] Jones DK, Knosche TR, Turner R (2013). White matter integrity, fiber count, and other fallacies: the do’s and don’ts of diffusion MRI. Neuroimage.

[14] Weber RA (2015). Diffusional kurtosis and diffusion tensor imaging reveal different time-sensitive stroke-induced microstructural changes. Stroke.

[15] Hui ES (2012). Stroke Assessment with Diffusional Kurtosis Imaging. Stroke.

[16] Cheung JS, Wang E, Lo EH, Sun PZ (2012). Stratification of heterogeneous diffusion MRI ischemic lesion with kurtosis imaging: evaluation of mean diffusion and kurtosis MRI mismatch in an animal model of transient focal ischemia. Stroke.

[17] Jensen JH, Helpern JA, Ramani A, Lu H, Kaczynski K (2005). Diffusional kurtosis imaging: the quantification of non-gaussian water diffusion by means of magnetic resonance imaging. Magn. Reson. Med..

[18] Umesh Rudrapatna S (2014). Can diffusion kurtosis imaging improve the sensitivity and specificity of detecting microstructural alterations in brain tissue chronically after experimental stroke? Comparisons with diffusion tensor imaging and histology. Neuroimage.

[19] Lu H, Jensen JH, Ramani A, Helpern JA (2006). Three-dimensional characterization of non-gaussian water diffusion in humans using diffusion kurtosis imaging. NMR Biomed..

[20] Hansen, B. & Jespersen, S. N. Recent developments in fast kurtosis imaging. *Frontiers in Physics* **5**, 10.3389/fphy.2017.00040 (2017).

[21] Jellison BJ (2004). Diffusion tensor imaging of cerebral white matter: a pictorial review of physics, fiber tract anatomy, and tumor imaging patterns. AJNR Am J Neuroradiol..

[22] Stieltjes B (2001). Diffusion tensor imaging and axonal tracking in the human brainstem. Neuroimage.

[23] Douek P, Turner R, Pekar J, Patronas N, Le Bihan D (1991). MR color mapping of myelin fiber orientation. J. Comput. Assist. Tomogr..

[24] Glenn GR, Helpern JA, Tabesh A, Jensen JH (2015). Optimization of white matter fiber tractography with diffusional kurtosis imaging. NMR Biomed..

[25] Skorpil M, Karlsson M, Nordell A (2004). Peripheral nerve diffusion tensor imaging. Magn. Reson. Imaging.

[26] Morisaki S (2011). *In vivo* assessment of peripheral nerve regeneration by diffusion tensor imaging. J. Magn. Reson. Imaging.

[27] Boyer RB (2015). 4.7-T diffusion tensor imaging of acute traumatic peripheral nerve injury. Neurosurg. Focus.

[28] Lehmann HC, Zhang J, Mori S, Sheikh KA (2010). Diffusion tensor imaging to assess axonal regeneration in peripheral nerves. Exp. Neurol..

[29] Yamasaki T (2015). *In vivo* evaluation of rabbit sciatic nerve regeneration with diffusion tensor imaging (DTI): correlations with histology and behavior. Magn. Reson. Imaging.

[30] Tannenbaum J, Bennett BT (2015). Russell and Burch’s 3Rs then and now: the need for clarity in definition and purpose. J. Am. Assoc. Lab Anim. Sci..

[31] Skorpil M, Engström M, Nordell A (2007). Diffusion-direction-dependent imaging: a novel MRI approach for peripheral nerve imaging. Magn. Reson. Imaging.

[32] Ozarslan E, Mareci TH (2003). Generalized diffusion tensor imaging and analytical relationships between diffusion tensor imaging and high angular resolution diffusion imaging. Magn. Reson. Med..

[33] Liu C, Bammer R, Acar B, Moseley ME (2004). Characterizing non-Gaussian diffusion by using generalized diffusion tensors. Magn. Reson. Med..

[34] Alexander AL (2011). Characterization of cerebral white matter properties using quantitative magnetic resonance imaging stains. Brain Connect..

[35] Feldman HM, Yeatman JD, Lee ES, Barde LH, Gaman-Bean S (2010). Diffusion tensor imaging: a review for pediatric researchers and clinicians. J. Dev. Behav. Pediatr..

[36] Heckel A (2015). Peripheral Nerve Diffusion Tensor Imaging: Assessment of Axon and Myelin Sheath Integrity. PLoS One.

[37] Alexander AL, Lee JE, Lazar M, Field AS (2007). Diffusion tensor imaging of the brain. Neurotherapeutics.

[38] Spampinato MV (2017). Diffusional Kurtosis Imaging and Motor Outcome in Acute Ischemic Stroke. AJNR Am. J. Neuroradiol..

[39] Winklewski, P. J. *et al*. Understanding the Physiopathology Behind Axial and Radial DiffusivityChanges—What Do We Know? *Front. Neurol*. **9**, 10.3389/fneur.2018.00092 (2018). 10.3389/fneur.2018.00092 PMC5835085 29535676

[40] Gaudet, A. D., Popovich, P. G. & Ramer, M. S. *In J Neuroinflammation* Vol. 8, 110 (2011). 10.1186/1742-2094-8-110 PMC3180276 21878126

[41] Menorca RM, Fussell TS, Elfar JC (2013). Nerve physiology: mechanisms of injury and recovery. Hand. Clin..

[42] Mori, S. & Tournier, J. D. *Introduction to diffusion tensor imaging and higher order models*. 2nd edition/edn, (Elsevier/Academic Press 2014).

[43] Fukunaga I (2013). Effects of diffusional kurtosis imaging parameters on diffusion quantification. Radiol. Phys. Technol..

[44] Jiang H, van Zijl PC, Kim J, Pearlson GD, Mori S (2006). DtiStudio: resource program for diffusion tensor computation and fiber bundle tracking. Comput. Methods Programs Biomed..

[45] Dyrby TB (2014). Interpolation of diffusion weighted imaging datasets. NeuroImage.

[46] Yeh FC, Verstynen TD, Wang Y, Fernández-Miranda JC, Tseng WY (2013). Deterministic diffusion fiber tracking improved by quantitative anisotropy. PLoS One.

[47] Veraart J (2011). More accurate estimation of diffusion tensor parameters using diffusion Kurtosis imaging. Magn. Reson. Med..

[48] Fieremans E, Jensen JH, Helpern JA (2011). White matter characterization with diffusional kurtosis imaging. Neuroimage.

[49] Farrell JA (2007). Effects of SNR on the Accuracy and Reproducibility of DTI-derived Fractional Anisotropy, Mean Diffusivity, and Principal Eigenvector Measurements at 1.5T. J. Magn. Reson. Imaging..

