# Supplementary material for: In vivo Diffusion Tensor Imaging, Diffusion Kurtosis Imaging, and Tractography of a Sciatic Nerve Injury Model in Rat at 9.4T
Source: Sci Rep. 2018 Aug 27;8:12911. doi: 10.1038/s41598-018-30961-1 (PMC6110718; doi:10.1038/s41598-018-30961-1)
Supplement: Supplementary file 1 — Supplementary Information [file 41598_2018_30961_MOESM1_ESM.pdf]

In vivo Diffusion Tensor Imaging, Diffusion Kurtosis Imaging, and Tractography of a Sciatic Nerve Injury Model in Rat at 9.4T

Gustav Andersson[1,2*], Greger Orädd[1], and Lev N. Novikov[1]

Supplementary Figure 1

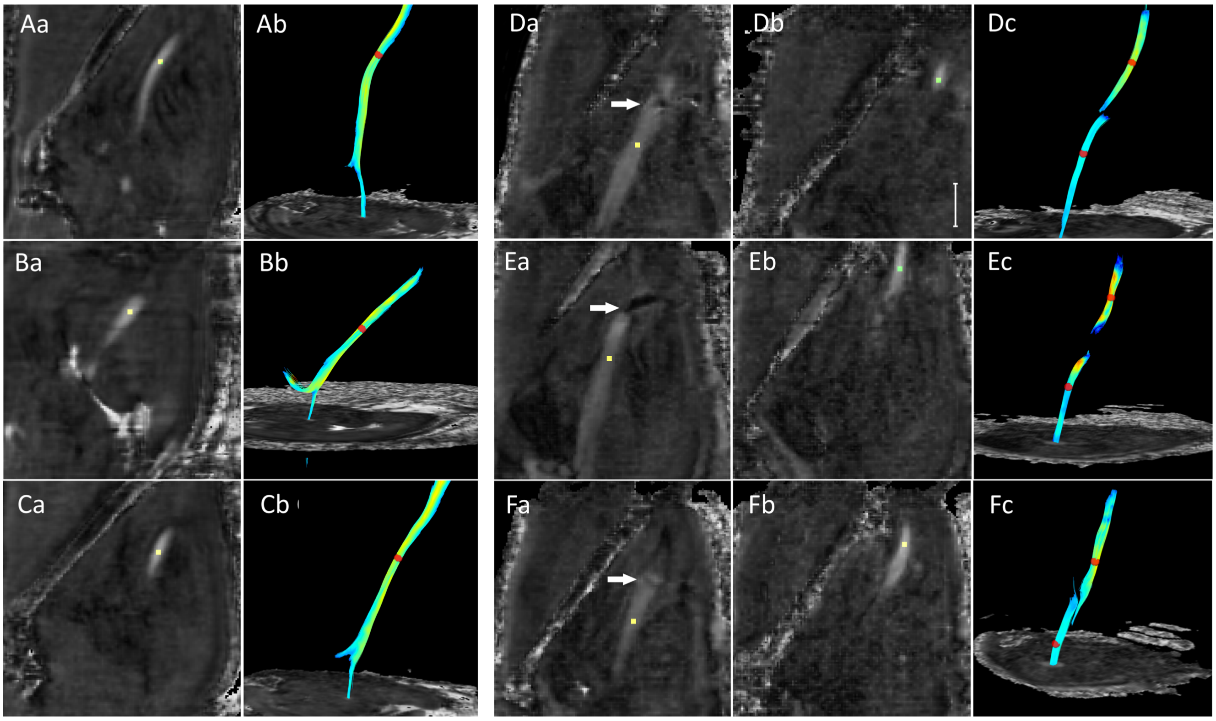

Multiple examples of FA greyscale slices in the sagittal orientation, with positioning of ROIs. In all images, the sciatic nerve is visible as the signal intensive structure marked with the ROI.
A-C correspond to preoperative scan with the ROI positioned in the sciatic nerve at 10 mm proximal to the sciatic nerve bifurcation into tibial and peroneal nerves.
D-F corresponds to postoperative scans: Da, Ea, Fa show the distal ROI placement at 5 mm distal to the nerve transection (arrow). Db, Eb, Fb show the proximal ROI placement in another slice, 5 mm proximally to the nerve transection.
Ab, Bb, Cb show the corresponding tractographies of the preoperative scans. Dc, Ec, Fc show the corresponding tractographies of the postoperative scans.
The dark structure to the left (ventral) of the nerve is the femur of the rat.

Supplementary Figure 2

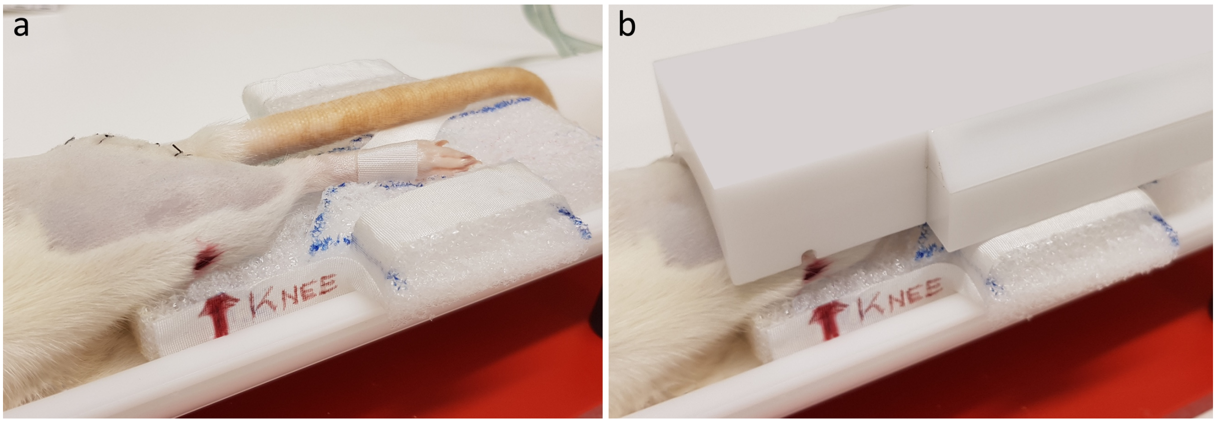

Positioning of the animal at the MR bed.
The animals were positioned in a right lateral decubitus position (a) with the brain array coil positioned over the left hip and thigh (b). A custom-made foam support was placed between the legs of the animal to minimize unwanted motion during the scans, and to achieve stable contact between the coil and the animal. Markings were made on the foam support in order to allow the animal and coil positioning to be recreated for all scans.
